# Atomic metal coordinated to nitrogen-doped carbon electrocatalysts for proton exchange membrane fuel cells: a perspective on progress, pitfalls and prospectives

**DOI:** 10.1039/d3ta04711c

**Published:** 2023-10-05

**Authors:** Angus Pedersen, Alexander Bagger, Jesús Barrio, Frédéric Maillard, Ifan E. L. Stephens, Maria-Magdalena Titirici

**Affiliations:** a Department of Materials, Royal School of Mines, Imperial College London London SW7 2AZ England UK a.pedersen19@imperial.ac.uk; b Department of Chemical Engineering, Imperial College London London SW7 2AZ England UK m.titirici@imperial.ac.uk; c Department of Physics, Technical University of Denmark Kongens Lyngby 2800 Denmark; d University Grenoble Alpes, University Savoie-Mont-Blanc, CNRS, Grenoble-INP, LEPMI 38000 Grenoble France; e Advanced Institute for Materials Research (WPI-AIMR), Tohoku University 2-1-1 Katahira, Aobaku Sendai Miyagi 980-8577 Japan

## Abstract

Proton exchange membrane fuel cells require reduced construction costs to improve commercial viability, which can be fueled by elimination of platinum as the O_2_ reduction electrocatalyst. The past 10 years has seen significant developments in synthesis, characterisation, and electrocatalytic performance of the most promising alternative electrocatalyst; single metal atoms coordinated to nitrogen-doped carbon (M-N-C). In this Perspective we recap some of the important achievements of M-N-Cs in the last decade, as well as discussing current knowledge gaps and future research directions for the community. We provide a new outlook on M-N-C stability and atomistic understanding with a set of original density functional theory simulations.

## Abstract 2

This Perspective highlights past achievements, knowledge gaps and future research directions for M-N-C catalysts for PEMFCs.

## Graphical abstract

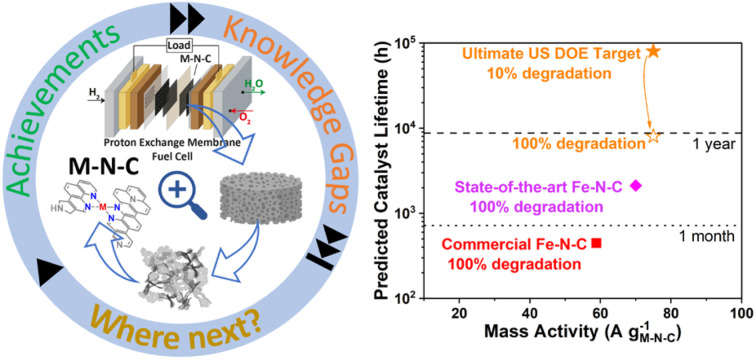

## Introduction

Low temperature proton exchange membrane fuel cells (PEMFCs) powered by green hydrogen offer high power density in stationary and transport application with zero CO_2_ emissions. One primary problem of PEMFCs is the kinetically sluggish cathodic O_2_ reduction reaction, which requires an efficient electrocatalyst to facilitate the reaction over 1000 s of hours. An ideal catalyst should have high turnover frequency (e^−^ site^−1^ s^−1^), accessible volumetric and gravimetric active site density (site cm^−3^ and site g^−1^) and stability (electrochemical turnover number, e^−^ site loss^−1^). Pt-based catalysts rank highest in all these categories for O_2_ reduction, hence their current commercial implementation. However, the US Department of Energy (DOE) identified that removing Pt from PEMFC cathodes will be key to achieving the ultimate PEMFC stack cost target of 30$ kW^−1^.^1^ Aside from cost, Pt is a critical and scarce material, with the vast majority mined in South Africa and Russia,^2^ where geopolitical tensions and national electricity shortages could lead to supply problems.

To date, the most promising alternative to Pt-based cathodes are those constructed from transition metal atoms coordinated to N-doped C (M-N-C, where M = metal).^3^ These single atom catalysts have the possibility of 100% active site utilisation and a binding energy that can be tuned according to the metal and local ligand environment. Those based on Fe-N-C, with an active site resembling the Fe-N_4_ site in heme, exhibit the highest activity for O_2_ reduction, approaching that of Pt.^4^ Nonetheless, they currently possess insufficient PEMFC stability for application in light duty automotive vehicles, where an 8000 h operation target has been set by the US DOE.^5^ The performance of M-N-C catalysts has been exhaustively covered by recent reviews by Osmieri *et al.*^6^ and Specchia *et al.*^7^ Meanwhile, the main synthesis and characterisation methods and currently known degradation pathways have been summarised by Asset *et al.*,^8^ Bae *et al.*^9^ and Kumar *et al.*^10^ A wider perspective on different single atom catalysts for various electrochemical applications has been provided by Cherevko and coworkers.^11^ Here, as part of the *Journal of Materials Chemistry A* 10-year anniversary, we aim to provide our perspective on important advancements and milestones in the past 10 years of M-N-C for PEMFCs. We also identify current critical knowledge gaps, including atomistic modelling, as well as future research directions, with a discussion of stability and its descriptors.

## 10 years of achievements

### Active site quantification

**Fig. 1:**
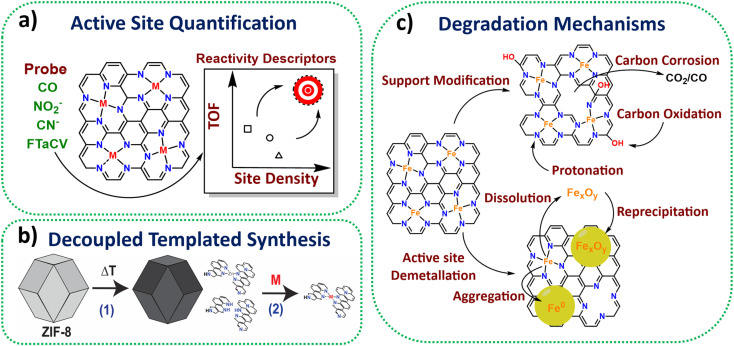
Schemes representing some highlighted achievements in method development and understanding in the past 10 years for M-N-Cs. (a) Active site quantification. (b) Decoupled synthesis consisting of pyrolysis of active site template materials, followed by active metal loading. (c) Identified degradation mechanisms of Fe-N-C.

A suite of *ex situ* (CO cryo adsorption^12^ and acid leaching^13^) and *in situ* electrochemical methods from half cell (cyanide^14^ and nitrite stripping^15^) to PEMFC (Fourier-transform alternating current voltammetry (FTacV)^16^) have been established for active site quantification in M-N-Cs (Fig. 1a). This has enabled comparison of turnover frequencies and establishment of reactivity descriptors among reported catalysts.

### Synthesis towards high density active sites

Zeolitic framework-8 (ZIF-8) has served as an ideal active site template to load active single metal atoms, due to its Zn-N_4_ based structure, which is stable to high temperatures (∼900 °C), therefore serving as an ideal active template. Several studies have recently used ZIF-8 to create high active site density Fe-N-C catalysts with state-of-the-art activity based on decoupled pyrolysis and metal loading techniques (Fig. 1b),^17,18^ originally proposed by Fellinger and coworkers.^19^

### Active site utilisation

While high density active sites are desired, they also need to be reactant-accessible to benefit performance. Several works have achieved 100% active site utilisation based on *ex situ* CO cryo adsorption, although numbers of sites quantified from electrochemical methods are lower, typically <10%.^20^

Wan *et al.* highlighted that electrochemical active utilisation (based on nitrite stripping) of ZIF-8 derived Fe-N-C could reach 40% at the lowest site density (1.3 × 10^19^ sites g_M-N-C_^−1^) using silica templating, although the utilisation fell with increasing site density.^21^ Attention has since turned to increasing the electrochemical active site utilisation and mass transport properties, while maintaining a high site density, by introducing more mesoporosity within the electrocatalyst, as shown in ours and others recent work.^22,23^ Alternative metal organic frameworks (MOFs) such as MIL-101 and MOF-5 have also been shown to possess improved mesoporosity compared to ZIF-8 upon pyrolysis, leading active M-N-Cs.^24,25^

### *Operando* and *in situ* characterisation and mechanisms

*In situ* and *operando* X-ray absorption spectroscopy (XAS) has been used successfully to monitor Fe-N-C redox behaviour.^26^*In situ* Mössbauer has enabled identification of two types of Fe-N-C active sites, being either highly active but unstable, or less active but more stable.^27^ Meanwhile, *operando* Mössbauer has recently been shown to identify the type of Fe-N-C site and a new intermediate.^28^*Operando* mass spectrometry methods have proved insightful in determining carbon oxidation and metal dissolution pathways and key degradation mechanisms (Fig. 1c),^29,30^ with this understanding improving catalyst stability by informing methods for reducing active site demetallation.^31^

### Stability

Commercialised M-N-C (discussed further below) has been reported to operate >500 h,^32^ although the reported activity was not comparable to Pt-based catalysts. Recently, an improvement in PEMFC stability (>300 h) of a high activity Fe-N-C, comparable to Pt/C, has been achieved by coating an atomically thin layer of N-C on a highly active Fe-N-C.^33^ Well dispersed nanoparticles (CeO_*x*_, TaO_*x*_) and single atom radical scavengers have also proved effective in reducing Fe-N-C degradation from reactive oxygen species.^34–36^ Reactivation of Fe-N-C to extend catalyst lifetime has also been successfully demonstrated through *in situ* electrochemical reduction which reduces O species,^37^ resulting in a short-lived improved turnover frequency.^38^

### Benchmark materials, standardized PEMFC protocols, and cross laboratory studies

For any technological progress it is critical standardized protocols and benchmark commercial materials are developed to ensure equipment and researchers can reproduce results. The recent introduction of these in the Fe-N-C community^39^ will lead to accelerated research progress. Meanwhile, cross laboratory studies have helped to understand the varying properties and reactivity descriptors of Fe-N-C catalysts synthesised *via* different methods.^20^

### Commercialisation

Commercial development of M-N-Cs has been undertaken by Pajarito Powder, Nisshinbo, and recently Celcibus AB. In 2017, Ballard Power Systems, through collaboration with Nisshinbo, announced the first commercial implementation of M-N-C catalysts for low power application of emergency power/wifi backpack.^32^ The application was well suited since M-N-C are known to be poison resistant to impurities and contaminants,^40^ which are likely present in emergency situations, such as fires and volcano eruptions. This signalled a breakthrough maturation of research and the prospect of striving towards large but more demanding applications, such as transport. The following sections outline current missing knowledge and hurdles that need to be overcome for further practical applications.

## Current knowledge gaps

### Life cycle assessment

**Fig. 2:**
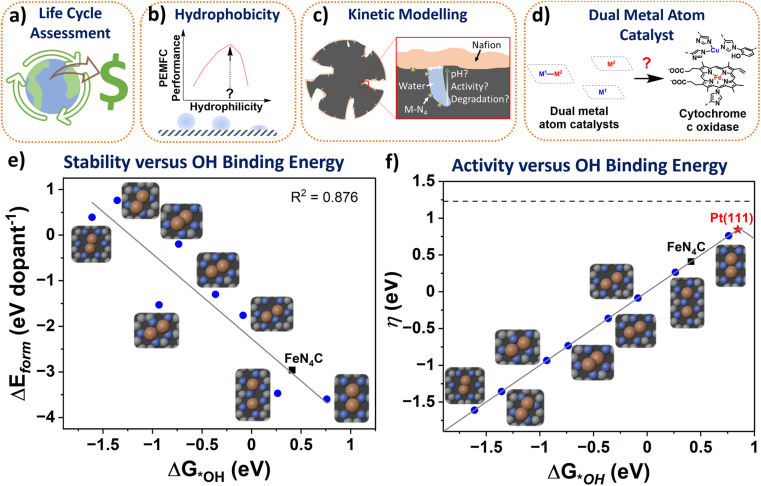
Schemes depicting knowledge gaps in the M-N-C literature. (a) Life Cycle Assessment and Monetized Impact. (b) Impact of hydrophobicity on PEMFC performance. (c) Representative kinetic modelling of active sites. (d) Dual metal atom catalysts mimicking enzymes for improved activity. (e) The formation energy of different dual site Fe_2_N_*x*_C (blue circles) and single atom FeN_4_C (black square) derived from Karmodak *et al.*^41^*versus* the calculated binding energy toward *OH calculated from DFT with revised Perdew–Burke–Ernzerhof functional. (f) The ORR volcano with Pt(111) at the top (red star), followed by the dual sites and reference FeN_4_C. ΔG_*OH_ is the hydroxyl adsorption energy. QV2 is the most stable dual site and predicted to be the most active. Colours in inset figures represent: Orange – Fe, Blue – Nitrogen, Grey – Carbon.

It is naturally assumed M-N-Cs have a lower environmental impact than Pt-based catalysts, with claims of environmentally benign synthesis,^42^ although this has yet to be shown or quantified *via* life cycle assessments. The answer may not be so trivial since state-of-the-art M-N-C catalyst typically incorporate multiple process steps and the quantities of wasted and required PEMFC components, including environmentally impactful Nafion, will be higher than for Pt-based catalysts. Also, the lifetime of state-of-the-art M-N-Cs is still far below that of Pt-based catalysts. Factoring environmental impacts from the life cycle assessment in technoeconomic analyses to date (Fig. 2a), could make M-N-C based PEMFC more (or less) economically competitive. Value stream mapping could also be integrated with life cycle assessments to ensure sustainable manufacturing and scalability (see section: Scalable).

### Translating from half-cell to PEMFC

Electrochemical half-cell systems do not accurately reflect degradation rates or mechanisms in real devices.^43^ This is unsurprising since degradation is a function of charge passed, where rotating disc electrodes operate at least an order of magnitude lower charge than a real device. For instance, a high-performance M-N-C at 0.6 V_RHE_ in O_2_-saturated 0.1 M HClO_4_ in a 1600 rpm rotating disc electrode (RDE) would expect *ca.* 5 mA cm^−2^, which corresponds to 25 A g_M-N-C_^−1^ at 0.2 mg_M-N-C_ cm^−2^. The same M-N-C at 0.6 V_RHE_ in an optimised H_2_–O_2_ PEMFC could then reasonably show 1 A cm^−2^ at 4 mg_M-N-C_ cm^−2^, equal to 250 A g_M-N-C_^−1^. Degradation rates and mechanisms also depend on temperature, where most half-cell tests are conducted at room temperature, rather than 80 °C as commonly used in PEMFCS.^44^ Critically, temperature affects the carbon oxidation reaction kinetics,^29,45^ which modifies electron delocalization and therefore turnover frequency of M-N-Cs.^37,38,46^ The quantitative effect of oxygen group chemistry on turnover frequency could be probed by high sensitivity temperature programmed desorption.

Considering other catalysts, a Mn–Co spinel alkaline O_2_ reduction catalyst exhibited improved activity under fuel cell conditions compared to half-cell.^47^ Meanwhile, various Pt nanoparticle shapes have been proven highly active in RDE, but their activity does not translate to fuel cell performance.^48^ For Fe-N-C, some have reported higher activity in RDE compared to PEMFC, taking into account temperature effects.^49,50^ Meanwhile previous work by Jaouen *et al.* found a *ca.* factor five higher activity for one Fe-N-C in PEMFC compared to RDE, again accounting for temperature affects. Although a separate Fe-N-C synthesised *via* a different route did not express such discrepancy. This difference for the former was attributed to anion adsorption on protonated N from the H_2_SO_4_ electrolyte in RDE.^51^ They also found optimisation of catalyst ink formulation was also crucial to obtain more comparative O_2_ reduction activities between RDE and PEMFC.^51^ Methods for catalyst layer optimisation in PEMFC are available for M-N-Cs,^52^ although the optimisation process can still be lengthy and trial-and-error based. Also, it should be considered the electrode preparation step can affect the M-N-C properties and consequent structure–activity–stability correlations.^53^ The change from double to triple phase boundary from half-cell to PEMFC can also impact properties such as active site utilisation and mass transport limitations. Changes in the microenvironment also impact reaction kinetics^54^ (see: Microenvironment and local pH).

### Fe-N-C redox and site density quantification

The reversible redox couple observed in some Fe-N-C catalysts at *ca.* 0.76 V_RHE_ in acidic medium has been extensively confirmed as the Fe(ii)/Fe(iii) transition from Fe–N_*x*_ sites,^26,27,55^ with some exploration of electrolyte effects.^56^ This redox has been ascribed to arise from high spin Fe^3+^ sites.^27^ Still, it remains unclear why some Fe-N-C catalysts, containing Fe^3+^–N_*x*_ sites as measured by *ex situ* Mössbauer, exhibit Fe redox in PEMFC but not in RDE. Several factors could be the cause, such as: the redox being masked by pseudocapacitance, Fe^3+^ sites charge being neutralised by an electrolyte anion in RDE and/or electrochemical inaccessibility of Fe^3+^ sites from catalyst structure and morphology effects. A rigorous study exploring these various effects is required since *in situ* electrochemical site quantification from FTacV relies on a detectable redox.^16^ Meanwhile, *in situ* nitrite stripping has experienced conflicting claims in the number of electrons transferred during the electrochemical process (3–5 e^−^).^15,57^ It could be that different M–N_*x*_ sites produce different products (hydroxylamine or ammonium) with a corresponding different number of electrons. This requires further investigation. Additionally, nitrite stripping has been reported to probe non-single metal atom species such as the N/C framework^58^ and iron oxides,^59^ where the latter makes post-mortem active site quantification non-trivial.

### Hydrophobicity

Surface hydrophobicity of the carbon support of M–N_*x*_ sites plays an important role in the water management and therefore performance of PEMFCs. However, water vapor physisorption measurements are rarely conducted in the M-N-C community, with some exploration for electrochemical CO_2_ reduction.^60^ Correlating hydrophobicity with PEMFC results (Fig. 2b) would help to identify optimum hydrophobicity for M-N-C performance and avoid deleterious flooding. Also, understanding how oxidation of the carbon support changes hydrophobicity over time would prove insightful to tuning PEMFC operation.

### Electronic conductivity

While it is obvious electronic conductivity plays an important role in all electrocatalysis, surprisingly few studies measure the electronic conductivity of electrocatalysts as prepared or when incorporated in devices as electrodes. Boettcher and coworkers have thoroughly investigated the effect of electronic conductivity on earth-abundant oxygen evolution catalyst performance;^61^ however limited work has been carried out on M-N-Cs.^62–64^ Experimentally measured through-plane electronic conductivity has been reported to be three orders of magnitude lower than the in-plane.^65^ Additionally, since the thickness of M-N-Cs is typically *ca.* one order of magnitude higher than Pt/C, the impact of electronic conductivity becomes significant. For instance, as noted by Kulikovsky, for typical carbon support electronic conductivity of 20 S m^−1^ with 100 μm catalyst layer thickness would lead to 50 mV loss.^62^ For the same catalyst thickness, Jaouen *et al.* modelled different proton and electronic conductivity values, finding 80 mV loss for electronic conductivity of 20 S m^−1^.^66^ They concluded electronic conductivity of 100 S m^−1^ would be required to limit losses at high current density.^66^

Jaouen and coworkers have also alluded to surface and local active site electronic conductivity effects.^46^ They suggested *operando* H_2_O_2_ generation which exfoliate graphene sheets or oxidise edges would decrease conductivity at graphene sheet edges, where active sites are preferentially located, potentially having a profound impact on turnover frequency.^46^ Expanding on previous work,^67^ understanding how concentrations and locations of O, N, active metal, surface area and pyrolysis temperature directly affect single carbon layers, particle, and bulk electronic conductivity would provide useful reference data. It would also be interesting to measure how conductivity differs *ex situ* and *in situ* and decreases over time with carbon corrosion and oxidation. This could then inform the subsequent effect on turnover frequency.^46^ Analysis of distribution of relaxation times from electrochemical impedance spectroscopy provides a complimentary pathway to deconvolute different effects of proton and electronic conductivity.^68^

### Microenvironment and local pH

As our understanding of electrocatalysis improves, focus is shifting to understanding the microenvironment.^69^ Local pH effects have been considered thoroughly in alkaline CO_2_ reduction,^70^ and is beginning wider attention in electrocatalysis;^71^ however this idea has so far received little attention in acidic O_2_ reduction degradation mechanisms.^72^ While PEMFCs operate in a strongly bulk acidic environment, the pH has been modelled to increases down small mesopore channels, which are not in direct contact with Nafion and are instead filled with water.^73^ Active sites of M-N-Cs are thought to reside within micropores,^20,74^ which are inaccessible to direct Nafion contact and are instead filled with water during PEMFC operation (Fig. 2c). It is worth noting high surface area microporous M-N-C materials contain 10–20 wt% water under atmospheric conditions.^19^ Therefore the consumption of protons and O_2_ at high current densities may lead to local pH changes at active sites, as recently explored in O_2_ reduction down to pH 2 in RDE.^75^

If local pH change does occur, this could have implications on the O_2_ reduction pathway, activity, and degradation route. For degradation pathway, considering the Pourbaix diagram of Fe, for certain potentials at increased pH, Fe^3+^ and Fe^2+^ form Fe_2_O_3_ species, which have been widely observed following stability tests.^27,59^ Meanwhile for activity, at pH 7, H^+^ is 10^−7^ M, which negatively affects kinetics.^76^

### Atomistic understanding

The binding energy of reaction intermediates to Pt nanoparticle can be modelled by density functional theory (DFT) with certain accuracy due to their crystalline nature, which can be mimicked by single crystal studies. To date, the best model M-N-C systems are macrocycles (*e.g.*, metal phthalocyanines or metal porphyrin),^77^ as these contain a well-defined structure in experiments and can be replicated atom by atom in quantum chemistry simulation. However, these macrocycles rapidly degrade or inactivate in acidic electrolyte. Additionally, the typical pyrolysis methods for making M-N-C catalysts create a variety of possible active sites with varying local carbon structures (defects, edges, oxygen groups), which modify the electron density. Another option is to accept the variety of sites in a pyrolysed catalyst and instead establish trends using one model, for example a M-N_4_-C site. Beyond establishing the atomic structure of the active site, the description of the electronic structures of this catalyst is not straight forward, as the M-N-C catalyst involve accessing multiple different magnetic spin-states. For example, prediction of the CO binding energy on these catalysts varies heavily with the exchange correlation functional.^78^ Additionally, modelling M-N-C for electrocatalysis encounter challenges of metal catalyst solvation effects and electrolyte effects.

### Dual metal atom active sites

We recently surveyed the literature on dual-metal atom catalysts.^79^ Enzymes and molecular catalysts based on dual metal atom sites have shown enhanced activity beyond state-of-the-art single atoms. Meanwhile, many reports suggest improved activity for catalysts putatively containing dual and single atom active sites compared to their equivalent materials containing just single atom sites. However, these catalysts still possess orders of magnitude lower turnover frequency than the enzymes they aim to mimic (Fig. 2d) and are not beyond state-of-the-art single atom catalysts. The reasoning for this remains unclear; perhaps dual metal atom sites produced to date are poisoned or unstable under reaction conditions,^80^ or are misidentified (see further discussion in section: Characterisation).

The stability and activitity of dual metal atoms has been investigated by the formation energy found from DFT simulations in a series of different dual and single atomic metal in nitrogen-doped carbon pockets by Karmodak *et al.*^41,81^ Here, we investigated the same structures, focussing on Fe_*x*_N_*y*_C sites, adding calculations of the binding energy of these sites to OH (Fig. 2e). Interestingly, a clear trend is observed between active site stability and OH binding energy, with less stable sites binding OH stronger. Dual metal atoms in hexa-vacancy sites could have a directed experimental synthesis, as we and others have reported, based on a C_2_N-derived structure.^82,83^ Although, it appears Fe_2_ hexa-vacancy sites are far less stable (Fig. 2e) and would become instantly oxidised by strong OH binding under reaction conditions. The most stable dual metal atom site under the modelled conditions was quad-vacancy, closely mimicking single atom FeN_4_C sites, but with an improved weakened OH binding, approaching activity of Pt(111) (Fig. 2f). However, controllably creating the dual metal atom quad-vacancy site experimentally does not appear straightforward.

It should also be considered for M-N-C sites, there is an upper limit of *ca.* 0.9 V_RHE_ in half cell (50 °C) before carbon oxidation begins.^29^ The upper operating limit on potential before debilitating carbon oxidation will occur is even lower in PEMFC conditions due to accelerated carbon corrosion kinetics at PEMFC operating temperatures of 80 °C.^30,84^ Consequently, the high activity at low overpotentials (<0.3 V) predicted by DFT for carbon-supported dual atom metal active sites^85,86^ would not be practical: at potentials positive of 1 V, the carbon support would corrode. Conceivably, the carbon could be stabilised if it were covered by an atomically thin and stable elemental layer, for instance, through atomic layer deposition.

## Where next?

### Stability

#### Predicting catalyst lifetime

**Fig. 3:**
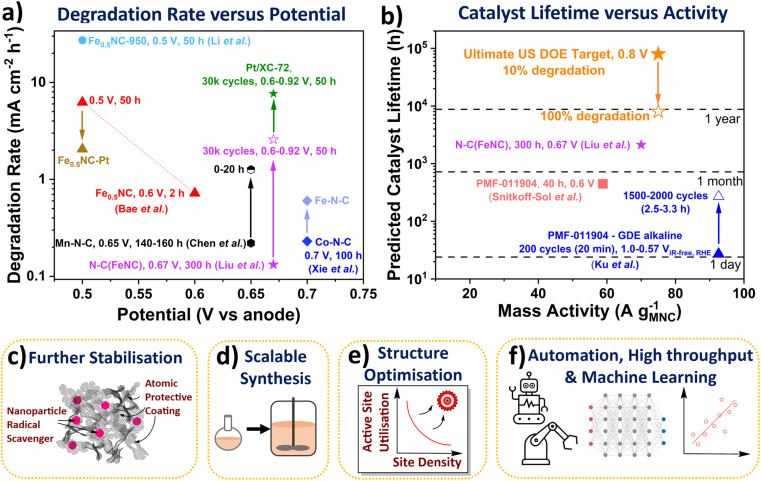
(a) Degradation rate of Fe-N-C and Pt/XC-72 catalysts in PEMFCs. Fuel cell potential represented *versus* hydrogen anode. All measurements were conducted at 80 °C. Liu *et al.*^33^ N-C(FeNC) 300 h at 0.67 V (H_2_/air 200 cm^3^ min^−1^) and 0.6 to ∼0.92 V with 3 s holds (30k cycles (50 h), H_2_/air 200 cm^3^ min^−1^, 1 bar_a_). Bae *et al.*^89^ Fe_0.5_NC 2 h potential hold at 0.6 V_vs anode_ and 50 h at 0.5 V_vs anode_. Li *et al.*^27^ Fe_0.5_NC-950 (H_2_/O_2_ 60 cm^3^ min^−1^, 1 bar_g_). Chen *et al.*^42^ Mn-N-C degradation rate between 140–160 h and 0–20 h holds at 0.65 V_vs anode_ (H_2_/air, 200 cm^3^ min^−1^, 1 bar_g_). Xie *et al.*^30^ Co-N-C and Fe-N-C (H_2_/air, 1 bar_g_). (b) Calculated catalyst lifetimes and measured mass activities. Ultimate US DOE light duty vehicle target of 8000 h (with 10% degradation) at 0.3 A cm^−2^ and 0.8 V_RHE_ (equivalent to 75 A g_M-N-C_^−1^ based on 4 mg_M-N-C_ cm^−2^ and 1 × 10^20^ sites g_M-N-C_^−1^). Snitkoff-Sol *et al.*^16^ Pajarito Powder FeNC (PMF 011904) 40 h at 0.6 V (H_2_/O_2_ 300 cm^3^ min^−1^, 1 bar_g_). Ku *et al.*^90^ PMF 011904 FeNC 1.0 to 0.57 V_iR-free RHE_ (0 to −125 mA cm^−2^) with 3 s holds (GDE, 0.1 M NaOH). Schemes representing future research directions. (c) Methods for improved stability. (d) Scalable synthesis. (e) Structure optimisation for increased active site utilisation. (f) Automation, high throughput and machine and deep learning.

Chronopotentiometry and accelerated stress test cycling enable quick comparison of degradation rates across different catalysts, as shown in Fig. 3a. These degradation rates can then be used to predict catalyst lifetime in PEMFCs, based on data-driven models.^87^ Although, as observed, the degradation rate is dependent on operation conditions (operating potential and hold *versus* cycling). It is therefore important to develop methods and descriptors, which can predict the lifetime of a catalyst based on a fundamental understanding at the active site level. For this, Cherevko and co-workers defined a stability number (electrochemical turnover number), which is applicable to all electrocatalysts and enables calculation of predicted catalyst lifetime (eqn (1)):^88^1
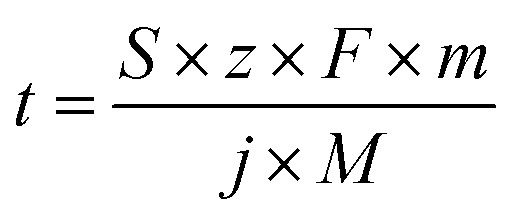
where *t* represents the lifetime of the active metal (s), *S* is the stability number, *z* is the number of electrons per O_2_ consumed (4), *F* is the Faraday constant (96 485 C mol^−1^), *m* is the mass of accessible active element (g cm^−2^), *j* is the applied current density (A cm^−2^), and *M* is the molar mass of the active element (g mol^−1^).

The stability number can be calculated from post-mortem Mössbauer and inductively coupled plasma mass spectrometry of the catalyst and/or electrolyte, or *via in situ* PEMFC techniques,^16^ or from lifetime extrapolated from degradation rates. These stability numbers can then be used to calculate the catalyst lifetime (Fig. 3b). A stability number of 3.37 × 10^8^ is required to reach the ultimate US DOE PEMFC target of 8000 h (with <10% degradation) at 0.3 A cm^−2^ and 0.8 V (equivalent to 75 A g^−1^ based on 4 mg_M-N-C_ cm^−2^ and 1 × 10^20^ sites g_M-N-C_^−1^).^5^ The stability number and lifetime of M-N-C in acidic electrolyte or PEMFC has not been reported to date, however it can be calculated, as mentioned above. Extrapolating state-of-the-art nitrogen-carbon coated Fe-N-C (N-C(Fe-N-C)) of Liu *et al.* shows lifetime of 2121 h at 0.67 V is feasible, with initial ∼70 A g^−1^. The catalyst lifetime can also be extrapolated from changes in the site density, as shown by Elbaz and coworkers.^16^ For a commercial Fe-N-C (Pajarito Powder PMF-011904), the catalyst lifetime was predicted to be 447 h in PEMFC at 58.8 A g^−1^ (based on 40 h hold at 0.6 V), with an apparently linear degradation rate of sites.^16^ Meanwhile, in alkaline GDE, Ku *et al.* measured the stability number changing from ∼10^6^ to ∼10^7^ for the same commercial Fe-N-C (PMF-011904), when evaluating after 200 and 2000 cycles (3 s hold at 1 and 0.57 V_iR-free, RHE_).^90^ This equated to catalyst lifetime changing from 27 h to 270 h at 92.6 A g^−1^.^90^ Bae *et al.* also found a decrease in degradation rate over the initial 2 h measurement for their Fe-N-C in PEMFC, with negligible site density loss beyond 1.5 h.^89^ This highlights the catalyst lifetime from the stability number is an overestimation of PEMFC operation since not all catalyst degradation routes directly alter the number of active sites (*e.g.*, attack from reactive oxygen species), and some activity recovery is also possible.^37^ It is therefore important to better understand differences between reversible and irreversible losses in PEMFCs. Additionally, decoupling contributions of different degradation mechanisms can identify critical parameters in M-N-C stability.^84^ Developing rates and descriptors for the different degradation mechanisms, while difficult, would be highly insightful for the community.

Theoretical tools for modelling material and active site stability with DFT have primarily been focused on thermodynamically deriving Pourbaix diagrams.^91,92^ Recent methods used for searching for acid stable oxygen electrocatalysts^93–95^ and metastable materials^96^ could be used for M-N-C catalyst to move beyond just the formation energies of the M-N-C sites. Noticeably, what the methods have in common is the starting point of simulating Pourbaix diagrams, which address the aqueous electrolyte potential and pH, but not the specific solvent species, such as anions and cations.

#### Improving long term stability

One of the biggest challenges of M-N-Cs is stabilising the inherently high surface energy of low coordinated single atoms. There is also the dilemma of intrinsically active catalysts typically possessing greater instability. After Fe-N-C, the next most active M-N-C candidates are Co-N-C and Mn-N-C, which benefit from their lower activity towards Fenton's reaction compared to Fe-N-C.^46,97^ They therefore exhibit less deactivation when contacted by H_2_O_2_. This has contributed to Co-N-C and Mn-N-C displaying 30–56% less degradation over PEMFC stability tests (16–100 h), compared to equivalently synthesised Fe-N-C.^30,42^ For the Co-N-C from Chen *et al.*,^42^ this difference in degradation can be visualised in Fig. 3a as a PEMFC degradation rate of 0.23 mA cm^−2^ h^−1^ compared to 0.60 mA cm^−2^ h^−1^ for equivalently synthesized Fe-N-C (held at 0.7 V for 100 h).

However, the degradation rate of this Co-N-C and a separate Mn-N-C (0.22 mA cm^−2^ h^−1^ after 160 h at 0.65 V)^30,42^ is greater than the recent state-of-the-art Fe-N-C by Liu *et al.* (0.13 mA cm^−2^ h^−1^ after 300 h at 0.67 V, labelled as (N-C)Fe-N-C in Fig. 3a).^33^ This Fe-N-C involved atomic N-C coating by chemical vapour deposition of an active Fe-N-C significantly enhanced stability, although with noticeable reduction in activity.^33^ The success of this method opens new avenues of research, including trying other potentially more stable coatings, and using more controlled deposition techniques for model studies, such as atomic layer deposition. Other alternatives to enhance stability could include modifying the catalyst support, for instance boron carbide has been previously used for Pt-based catalyst.^98^ Also, as mentioned earlier, nanoparticle radical scavengers have proved effective in minimising oxidative attack (Fig. 3c), although these nanoparticles could block active sites and may not be situated atomically close to active sites. More desirable scavengers would be single atoms scavengers doped within the catalyst support that are within proximity to the active site, due to the short lifetime of reactive oxygen species.^35^ Secondary metal atoms are often doped within M-N-C which improve activity and stability, although the exact reason for improved performance is only beginning to be elucidated.^99^ Pt atoms have been successfully incorporated into Fe-N-C with the performance improvement understand,^89^ although cheaper dopants are more desirable in future.

Aside from improving the degradation resistance of the catalyst, PEMFC operating conditions can also be tuned to minimize catalyst degradation. This can be achieved through model development, which identify key degradation parameters, as completed for a Pt-based catalyst.^100^

#### Reporting and standardization

As recently highlighted by Smith and Dickinson,^101^ many reports do not include sufficient experimental details to reproduce results, especially in electrochemical reporting. This slows the progress of research and knowledge dissemination. Publishers can improve electrocatalyst measurement reporting by establishing submission guidelines, as recently set for all American Chemical Society journals.^102^ Templates for synthesis and reporting would also help to ensure standardization and reproducibility between labs, which can be helped by automated high throughput development.

#### Gas diffusion electrode

Several gas diffusion electrode architectures have been developed which can probe catalyst performance and stability under device relevant high current conditions with less system complexity than PEMFCs.^103^ Studies involving GDEs with M-N-Cs in alkaline have recently become more widespread, although surprisingly few studies have implemented M-N-Cs in acidic GDE environments.^104^ We expect GDE tests will be more frequently utilized to accelerate electrocatalyst development and bridge across the “valley of death”.^105^ However, the use of electrolyte in GDEs still poses problems in fully representing PEMFCs. The electrolyte could result variation in pH across the catalyst layer, which requires modelling investigation.

### Synthesis

#### Pyrolysis

The main synthesis restriction with M-N-Cs to date is their pyrolysis step, which creates conductivity and activates the N-C scaffold. However, the resulting material displays a somewhat random arrangement and distribution of atomic moieties, which limits atomic control of the active site. An intrinsically electronically conductive MOF with a crystal structures containing Ni-N_4_ sites has shown high activity towards H_2_O_2_ production without a pyrolysis step.^106^ Although, this was only realised once mass transport limitation was removed.^106^ Future directions can therefore look to improve mass transport in such MOF materials and tune selectivity to H_2_O production for PEMFCs. These well-defined crystalline catalysts will also enable fundamental and model studies, which have not been possible to date.

Still, these non-pyrolysed MOF-based materials do not represent the class of M-N-C. M-N-Cs would benefit from better understanding of the “black box” pyrolysis step. Comprehensive *in situ* pyrolysis studies have been limited in M-N-Cs, mainly led by Zenyuk and coworkers.^107,108^

#### Scalable

The precursors used to manufacture M-N-Cs are orders of magnitude cheaper than Pt-precursors; however, scalability is largely influenced by the number and type of process steps. One-pot synthesis methods do not reach state-of-the-art performance, on the other hand the most promising catalyst synthesis protocols for enhanced stability typically involve decoupled pyrolysis and metal loading steps, with additional reaction steps. Often this includes an acid washing step, which is predicted to account for 49% of the catalyst manufacturing cost when produced at scale (500 000 PEMFC stacks year^−1^).^109^ Future directions of state-of-the-art M-N-C should therefore consider scalable routes (Fig. 3d), with minimal number of reaction steps and environmental impact, avoiding acid leaching steps where possible for commercialisation.

#### High active site utilisation with high active site density

As highlighted earlier, significant steps have been taken to improve active site density and active site utilization; however, achieving both simultaneously has remained elusive (Fig. 3e). This is mainly due to most high active site density catalysts being derived from ZIF-8, which is a microporous particle that limits electrochemical accessibility of active sites. Ionothermal templating methods have been proven effective, for instance by rendering highly mesoporous N-C substrates that led to electrochemical active site utilization >50% based on *in situ* nitrite stripping (5 e^−^ process assumed).^22^ Nevertheless, such high utilization arises partly owing to the low metal loading (∼0.5 wt%). Fine tailoring nitrogen content in templated N-C materials to allow a higher number of binding sites without reducing the electronic conductivity of the support will provide a suitable compromise between high active site density and utilization.

#### Fuel cell

For high performance M-N-C-based PEMFCs, the catalyst active site and catalyst layer design and morphology need to be considered concomitantly. PEMFC performance reports have mainly focussed on US DOE activity targets of 0.044 A cm^−2^ at 0.9 V_RHE, *iR*-free_.^5^ For practical applications, the US DOE activity target of 0.3 A cm^−2^ at 0.8 V_RHE_ is more critical to achieve.

The lower mass activity of M-N-C compared to Pt-based catalysts necessitates ×10 thicker catalyst layers in PEMFCs to feasibly achieve high current demands. Further research is therefore required for designing thick electrodes with optimised pore structures and properties that minimise losses in proton and electronic conductivity, while maintaining sufficient O_2_ accessibility. Nature could provide inspiration for the catalyst layer design, as we have recently highlighted,^110^ and new architectures such as grooving,^111^ could be explored for M-N-Cs. Methods for thick electrode engineering, manufacturing and characterisation can also be adapted from battery literature.^112^ Meanwhile, better understanding of gas and water flow in thick M-N-C cathode layers is required to understand and optimise PEMFC performance. This could be realised through multi-phase modelling with deep learning, as recently shown for a Pt-based PEMFC system.^113^

#### High throughput, deep learning and machine learning

Acceleration of research and discovery is required to overcome the looming societal challenges. Tools to assist researchers are becoming more widespread (Fig. 3f). For instance, high throughput synthesis and machine learning has recently been demonstrated for optimisation of Fe-N-C synthesis parameters.^114,115^ Machine learning can be used as a PEMFC diagnostic,^116^ whereas deep learning can also be applied for degradation prediction.^87^ Accumulation of high quality electrochemical and characterisation data sets with algorithms for analysis are required for best use of these tools.

While M-N-Cs are relatively restricted systems, the combined chemistry, material, and engineering space are so vast that traditional trial-and-error, high throughput and DFT methods are insufficient for exploration. Different approaches based on artificial intelligence could probe the space from a different perspective. Automation of characterisation, especially in microscopy, is also a growing direction.^117^

Recent studies on high entropy alloys have driven high throughput experiments coupled with machine learning can both refine our atomistic understanding of oxygen reduction activity and lead to the discovery of new catalyst materials;^118^ we envisage that such data driven hypothesis-based approaches could lead to even more active materials in future.^119^

#### Characterisation

Still, a bottle neck in processing novel M-N-C is their unequivocal characterisation, where national synchrotron and special microscopy facilities are required. For instance, we recently reported an approach towards Fe_2_ atoms in a C_2_N-like framework, based on high angle annual dark field scanning transmission electron microscopy and XAS.^82^ Even then, there is difficulty to differentiate sites, and is open to interpretation. As we later found, a repeated XAS measurement and analysis resulted in a better fit for a penta-coordinated single atom FeN_*x*_ site.^120^ Mössbauer spectroscopy is ideal for Fe-N-C characterisation and has aided their development, but enriched ^57^Fe is typically required and this technique is not applicable to other promising M-N-C such as those based on Mn or Co. Atomic resolution laboratory-based characterisation techniques which are more freely accessible to the wider community would help to accelerate research progress and resolve structure–activity trends.

Alternatively, increased active site densities will allow use of techniques which were previously insensitive due to their limit of detection. For instance, developments in laboratory-based X-ray absorption equipment, such as from EasyXAFS LLC, may be applicable for some high loading single atom M-N-Cs. Meanwhile, laboratory-based X-ray pair distribution function (XPDF) represents an under-utilised technique in the community, which could provide spatial resolution of the local atomic arrangement within M-N-Cs and long range order changes,^121^ and requires further exploration.

However, the community should be careful drawing conclusions from bulk characterisation techniques. As we discussed earlier in the section: Active site utilization, the vast majority (70–90%) of active sites in M-N-C are typically not electrochemically utilized. Therefore, operando characterisation techniques which probe the bulk M-N-C layers, such as Mössbauer spectroscopy, XAS or XPDF, will measure accessible and inaccessible active sites. This makes it difficult to deconvolute the contribution of activity and stability of different M–N_*x*_ sites. This challenge could be overcome by coupling *in situ* electrochemical probing techniques, such as FTacV, with bulk operando techniques.

For surface sensitive characterisation, time-of-flight secondary ion mass spectrometry (ToF-SIMS) is technique which has rarely been used. It can provide atomic surface chemical compositions of M-N-Cs, including light elements all the way up to hydrogen, with capabilities of depth profiling. ToF-SIMS can also distinguish single and dual metal atoms;^122,123^ however, quantification using ToF-SIMS is highly challenging.

#### Applications beyond oxygen reduction and electrocatalysis

The vast research undertaken on M-N-C catalysts for O_2_ reduction provides an extensive library of well characterised materials for other fields to “stand on the shoulders of giants”. Recent applications of M-N-C beyond other electrocatalytic reactions (CO_2_ reduction to CO) include thermocatalytic aerobic oxidation^124^ and hydrogenation,^125^ and enzyme mimicking for synthetic drug exploration.^126^ Perhaps commercial opportunities for M-N-Cs lie beyond the target of PEMFCs.

## Conclusions

In this Perspective we highlight significant steps taken by the community in improving knowledge of M-N-C electrocatalysts for PEMFCs over the last 10 years. Still, many stones remain unturned, and hurdles prevent further commercial M-N-C implementation. To this end, we discuss some of these unexplored research questions and future challenges. We present original DFT simulations which show correlation between stability (formation energy) and activity (OH binding energy) of dual atom Fe-N-C sites. We also highlight new perspectives on M-N-C stability *via* catalyst lifetime calculations, which illustrates the gap from state-of-the-art to reach ultimate US DOE targets.

## Methods

### Density functional theory

The atomic M-N-C structures and formation energy were obtained from Karmodak *et al.*, assuming a single sheet of graphene as the surrounding carbon structure.^41^ Here, an *OH intermediate was added to the structures and spin polarised for the clean and *OH structures. For relaxation computations GPAW code was used,^127,128^ with the following settings: (4, 4, 1) *k*-point, a grid spacing of 0.18, with the BEEF-vdW functional,^129^ and relax the structures to below 0.05 eV per atom. After relaxing the structures, the OH binding energy was calculated from the following equation:
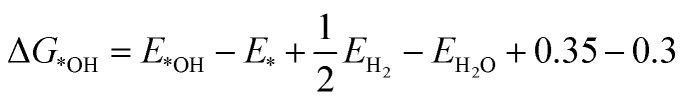
Here, 0.35 eV and −0.3 eV are thermodynamic and water stabilization corrections, respectively, obtained from Nørskov *et al.*^130^

## Author contributions

A. P. wrote the initial draft. J. B. and A. B. assisted writing the original draft. A. B. carried out DFT simulations. F. M., I. E. L. S. and M.-M. T provided supervision, and revised and edited the manuscript.

## Conflicts of interest

There are no conflicts to declare.

## Supplementary Material
